# Streptozotocin Induces Alzheimer’s Disease-Like Pathology in Hippocampal Neuronal Cells *via* CDK5/Drp1-Mediated Mitochondrial Fragmentation

**DOI:** 10.3389/fncel.2020.00235

**Published:** 2020-08-04

**Authors:** Junghyung Park, Jinyoung Won, Jincheol Seo, Hyeon-Gu Yeo, Keonwoo Kim, Yu Gyeong Kim, Chang-Yeop Jeon, Min Kyoung Kam, Young-Hyun Kim, Jae-Won Huh, Sang-Rae Lee, Dong-Seok Lee, Youngjeon Lee

**Affiliations:** ^1^National Primate Research Center, Korea Research Institute of Bioscience and Biotechnology (KRIBB), Cheongju, South Korea; ^2^Department of Functional Genomics, KRIBB School of Bioscience, Korea University of Science and Technology, Daejeon, South Korea; ^3^School of Life Sciences, BK21 Plus KNU Creative BioResearch Group, Kyungpook National University, Daegu, South Korea

**Keywords:** Alzheimer’s disease (AD), streptozotocin (STZ), hippocampus, mitochondrial dynamics, dynamin-1-like protein (Drp1), cyclin-dependent kinase 5 (CDK5)

## Abstract

Aberrant brain insulin signaling plays a critical role in the pathology of Alzheimer’s disease (AD). Mitochondrial dysfunction plays a role in the progression of AD, with excessive mitochondrial fission in the hippocampus being one of the pathological mechanisms of AD. However, the molecular mechanisms underlying the progression of AD and mitochondrial fragmentation induced by aberrant brain insulin signaling in the hippocampal neurons are poorly understood. Therefore, we investigated the molecular mechanistic signaling associated with mitochondrial dynamics using streptozotocin (STZ), a diabetogenic compound, in the hippocampus cell line, HT-22 cells. In this metabolic dysfunctional cellular model, hallmarks of AD such as neuronal apoptosis, synaptic loss, and tau hyper-phosphorylation are induced by STZ. We found that in the mitochondrial fission protein Drp1, phosphorylation is increased in STZ-treated HT-22 cells. We also determined that inhibition of mitochondrial fragmentation suppresses STZ-induced AD-like pathology. Furthermore, we found that phosphorylation of Drp1 was induced by CDK5, and inhibition of CDK5 suppresses STZ-induced mitochondrial fragmentation and AD-like pathology. Therefore, these findings indicate that mitochondrial morphology and functional regulation may be a strategy of potential therapeutic for treating abnormal metabolic functions associated with the pathogenesis of AD.

## Introduction

Alzheimer’s disease (AD) is the most common neurodegenerative disease and is characterized by the continuing loss of memory, cognitive declines, and atrophy of cortical and hippocampus of the brain. Typical pathological hallmarks of AD include the accumulation of amyloid-β (Aβ) plaques and the formation of neurofibrillary tangles in the cerebral cortex and hippocampus (Kesslak et al., 1991; Querfurth and LaFerla, 2010). Emerging evidence has indicated that insulin signaling is closely involved in numerous brain functions, including energy metabolism, neural plasticity, and cognition. Therefore, cerebral glucose metabolic dysfunction and deregulated brain insulin signaling in the brain has also been recognized as an early sign of sporadic AD (sAD; Grieb, 2016).

Streptozotocin (STZ) is a diabetogenic compound and is generally used to determine experimental animal models of diabetes due to its selective impairment activity of the insulin signal pathway (Lee et al., 1983). Previous studies have shown that intracerebroventricular (ICV) administration of low STZ doses into the brain induces disrupted homeostasis of brain insulin signaling and defect in cerebral glucose metabolism. This is accompanied by neuropathological and biochemical changes similar to those observed in the pathology of sAD such as synaptic dysfunction, tau hyperphosphorylation, oxidative stress, and neuronal loss through reduced expression of the insulin receptor (IR), thereby resulting in the desensitization of IRs without inducing a systemic diabetic condition (Salkovic-Petrisic and Hoyer, 2007). However, the precise molecular signaling pathway in the sAD model induced by STZ is still unclear.

Mitochondria are essential cellular organelles with key regulatory functions in energy production, oxidative balance, and calcium homeostasis. Mitochondria are especially important in the brain since neurons have a high demand for functional mitochondria to supply their high energy requirement, mainly for synaptic processes, and therefore neurons are vulnerable to mitochondrial deficiency (Du et al., 2012; Lin and Sheng, 2015). The brains of patients with AD also show several molecular abnormalities, such as loss of energy metabolism and oxidative stress (Reddy, 2009; Rhein et al., 2009; Verri et al., 2012). In addition to the STZ-induced AD model, mitochondrial abnormalities such as abnormal morphology, decrease adenosine triphosphate (ATP) biosynthesis, and accumulation of reactive oxygen species (ROS) were observed (Guo et al., 2017). Therefore, more details regarding the molecular mechanisms of STZ-induced mitochondrial dysfunction are needed for the development of therapeutic strategies for treating patients with AD.

Mitochondria are highly dynamic subcellular organelles that repeat the process of fusion and fission with each other. Mitochondrial fusion is regulated by optic atrophy 1 (Opa1), mitofusin 1 (Mfn1), and mitofusin 2 (Mfn2), and mitochondrial fission is regulated by dynamin-1-like protein (Drp1) and mitochondrial fission 1 (Fis1; Knott et al., 2008; Cho et al., 2010; Westermann, 2010). Mitochondrial dynamics, an equilibrium of mitochondrial fission and fusion, is crucial for cellular processes, such as mitochondrial biogenesis, mitochondrial disassembly, mitochondrial distribution and transport, and regulation of cell division and cell death (Cho et al., 2010). Imbalances of mitochondrial dynamics in neuronal cells trigger mitochondrial dysfunction, subsequently leading to the reduction of mitochondrial membrane potential, ATP depletion, ROS accumulation, and increase of apoptosis (Wu et al., 2011; Park et al., 2015a; Ruiz et al., 2018). Drp1, a key regulator of mitochondrial fission, is translocated from the cytosol to the mitochondria by the increased of its activity. The accumulation of mitochondrial fission induces mitochondrial fragmentation, and it is closely associated with various neurodegenerative disorders (Rappold et al., 2014; Filichia et al., 2016). The activity of Drp1 is tightly controlled by post-translational modifications including phosphorylation (Elgass et al., 2013). Especially, phosphorylation of Ser616 (S616) results in increased activity of Drp1, which plays an important role in various neurodegenerative pathological processes including AD (Wang et al., 2009; Bradshaw et al., 2016; Prieto et al., 2016). Moreover, previous studies have demonstrated that mitochondrial abnormalities played a role in the STZ-induced rat model of sAD (Correia et al., 2013; Yang et al., 2014). However, the molecular relationship between changes in mitochondrial dynamics and STZ-induced sAD has not been fully elucidated.

Cyclin-dependent kinase 5 (CDK5) is a proline-directed serine/threonine kinase that performs an important role in the maintenance of synaptic functions and survival of neurons (Dhavan and Tsai, 2001; Shah and Lahiri, 2014). Although CDK5 is ubiquitously expressed, it is mainly controlled by p35, which is activated after being cleaved into p25, resulting in hyperactivity of CDK5 (Tsai et al., 1994; Tang et al., 1995). Inappropriate activation of CDK5 plays an early role in the process of neuronal death, even before the mitochondrial dysfunction. Therefore, maintenance of CDK5 homeostasis is suggested as a reasonable therapeutic target for ameliorating AD pathological processes, such as neuronal apoptosis and tau pathology (Sun et al., 2008; Lopes et al., 2010; Piedrahita et al., 2010). Interestingly, CDK5 was identified as an upstream regulator of mitochondrial fission *via* phosphorylation of Drp1 at S616 in neurodegenerative conditions (Meuer et al., 2007; Cherubini et al., 2015; Jahani-Asl et al., 2015). However, the mechanisms *via* which CDK5 regulates mitochondrial fission by phosphorylation of Drp1 at S616 during the hippocampal loss in AD are still not fully understood.

To validate the precise molecular signaling in the sAD-like model induced by STZ, we explored the changes in neuronal apoptosis, synaptic function, tau pathology, and mitochondrial morphology in STZ-induced hippocampal neuronal HT-22 cells.

## Materials and Methods

### Cell Culture and Treatment

HT-22 cells were derived from HT-4 cells, which were immortalized from primary mouse hippocampal neuronal culture (Davis and Maher, 1994). HT-22 cells were maintained at 37°C in DMEM with high glucose (Welgene, Daegu, Korea) supplemented with 10% FBS (Thermo Fisher Scientific, Waltham, MA, USA), 100 U/ml penicillin, and 100 μg/ml streptomycin (Welgene) in a humidified atmosphere incubator (Thermo Fisher Scientific, Waltham, MA, USA) with 5% CO_2_. The cells were pretreated with U0126 (10 μM; Sigma–Aldrich, St. Louis, MO, USA), Mdivi-1 (10 μM; Sigma–Adrich, St. Louis, MO, USA), and roscovitine (10 μM; Sigma–Adrich, St. Louis, MO, USA) for 30 min and were incubated with STZ (10 mM).

### MTT Assay

HT-22 cells were cultured on 96-well plates for 24 h. Next, HT-22 cells were incubated for 30 min at 37°C with MTT (0.5 mg/ml; Sigma–Adrich, St. Louis, MO, USA), and then 100 μl DMSO (Sigma–Adrich, St. Louis, MO, USA) was added. Absorbance was measured at 550 nm.

### Western Blot Analysis

Whole protein lysates were prepared using the PRO-PREP protein extraction solution (Intron Biotechnology, Seongnam, Korea), and mitochondrial and cytoplasmic fractions were performed with a mitochondria isolation kit (Thermo Fisher Scientific, Waltham, MA, USA). Equal amounts of proteins were separated by electrophoresis on 8–12% SDS-PAGE gels and transferred onto nitrocellulose membranes (BD Biosciences, San Jose, NJ, USA). The membranes were blocked by incubation in blocking buffer (BD Biosciences) and probed with the following antibodies overnight at 4°C: anti-NeuN, anti-AT8, anti-Tau-5, anti-β-actin (Sigma–Adrich, St. Louis, MO, USA), anti-PSD95, anti-phospho(p)-Tau(T181), anti-p-Tau(S396; Abcam, MA, USA), anti-Drp1, anti-p-Drp1(S616), anti-COXIV, anti-GAPDH, anti-PARP, anti-cleaved caspase-3, p-Tau(S262), anti-CDK5, anti-ERK, anti-p-ERK, anti-GSK3β, anti-p-GSK3β(S9; Cell Signaling, MA, USA), and anti-p35 (Thermo Fisher Scientific, Waltham, MA, USA). The membranes were washed with TBS with 0.1% Tween-20 (TBST) and incubated with horseradish peroxidase-conjugated secondary antibodies (Cell Signaling) for 1 h at room temperature. After washing with TBST, specific binding was detected using a chemiluminescence detection system (Thermo Fisher Scientific, Waltham, MA, USA).

### Preparation of Stable Cell Lines and Mitochondrial Imaging

DsRed2-mito including plasmid (pLenti6.3-DsRed2-mito) was kindly provided by Dr. Dong-Seok Lee (Kyungpook National University, Daegu, Korea). pLenti6.3-DsRed2-mito plasmid was transfected into HT-22 cells by using effective (Qiagen, CA, USA) according to the manufacturer’s instructions. After 24 h, the transfected cells were selected using 8 μg/mL blasticidin (Thermo Fisher Scientific, Waltham, MA, USA). DsRed2-mito expressing HT-22 cells were seeded on 0.01% poly-D-lysine-coated round coverslips and were incubated for 24 h. After experiments, cells were washed with PBS and fixed with 4% paraformaldehyde for 1 h. After washing, the coverslips were mounted on slides with mounting medium (VECTOR Laboratories, CA, USA). Mitochondrial images were acquired using the LSM-710 confocal microscope (Carl Zeiss, Jena, Germany). Mitochondrial length measurements were performed using ImageJ software as previously described (Park et al., 2013).

### Measurements of Intracellular ATP Levels

The intracellular ATP levels were determined by using an ATP determination kit (Thermo Fisher Scientific, Waltham, MA, USA) following the manufacturer’s instructions. Whole protein lysates at a concentration of 1 μg/ml were used for the measurements of intracellular ATP levels.

### Measurements of Intracellular ROS

Intracellular ROS generation was assessed using CM-H2DCFDA. HT-22 cells were incubated with 5 μM CM-H2DCFDA (Thermo Fisher Scientific, Waltham, MA, USA) for 30 min at 37°C and then analyzed by FACSCalibur flow cytometry (BD Biosciences).

### Statistical Analysis

The data represent the mean and SD from three independent experiments (*n* ≥ 3). Experimental differences were tested for statistical significance using two-way ANOVA using the GraphPad Prism 5 software (San Diego, CA, USA). A p-value < 0.05 was deemed to be statistically significant and is indicated on graphs by an asterisk. P-values < 0.01 and < 0.001 are indicated by two and three asterisks, respectively.

## Results

### STZ-Induced Neurotoxicity and Apoptotic Cell Death in HT-22 Cells

**Figure 1:**
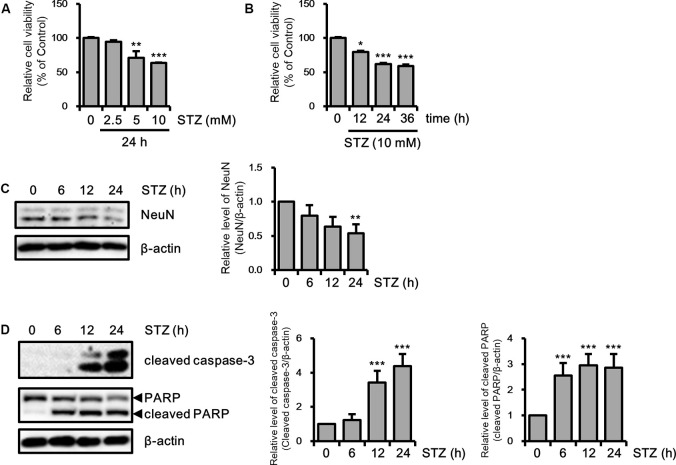
Effect of streptozotocin (STZ) on apoptotic neuronal loss in HT-22 cells. **(A)** Dose-dependent changes in cell viability were confirmed using the MTT assay with the indicated STZ concentration (2.5–10 mM) for 24 h. **(B)** HT-22 cell viability was determined using the MTT assay according to STZ (10 mM) treatment with the indicated time points (6–24 h). **(C)** The protein expression level of hexaribonucleotide Binding Protein-3 (NeuN) was determined by western blotting in STZ-induced HT-22 cells. **(D)** Cleaved caspase-3 and cleaved PARP were confirmed by western blotting. The data are presented as mean values ± SD (*n* ≥ 3). *p < 0.05, **p < 0.01, and ***p < 0.001.

Because previous studies have investigated neuronal toxicity by STZ in neuroblastoma cells (Biswas et al., 2016; Guo et al., 2017), we determined the neurotoxicity of STZ on HT-22 cells in a dose- and/or time-dependent manner using the MTT assay. Cell viability was decreased to approximately 60% at 10 mM of STZ treatment for 24 h (Figures 1A,B). We confirmed the STZ effect on neuronal loss in HT-22 cells with NeuN, a neuronal marker. Results showed that the expression level of NeuN was reduced in a time-dependent manner (Figure 1C). Furthermore, we determined the effect of STZ on apoptotic cell death by verifying the alterations level of cleaved caspase-3 and cleaved PARP. Our result indicated that the level of cleaved caspase-3 and cleaved PARP increased by STZ treatment (Figure 1D). These results suggested that STZ induced apoptotic neuronal loss in hippocampus cells.

### Effect of STZ on the sAD-Like Pathology in HT-22 Cells

**Figure 2:**
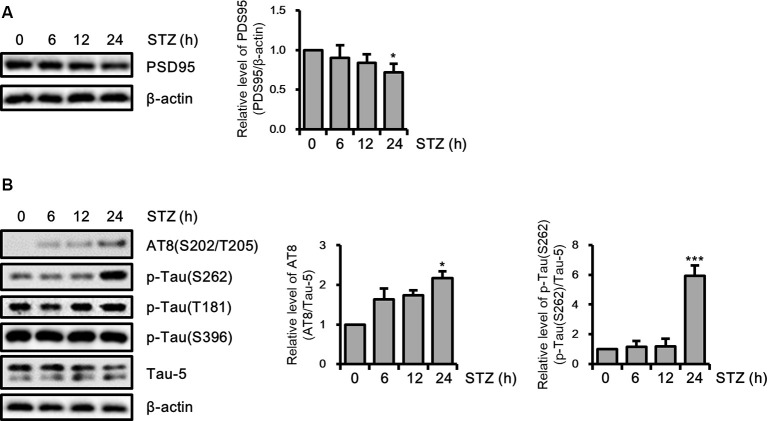
Effect of streptozotocin (STZ) on synaptic loss and tau activation in HT-22 cells. **(A)** PSD95 was confirmed by western blotting. **(B)** The expression levels of AT-8, p-Tau(S262), p-Tau(T181), and p-Tau(S396) in STZ-treated HT-22 cells for the indicated times were verified with western blot analysis. Tau-5 was the loading control of AT-8, p-Tau(S262), p-Tau(T181), and p-Tau(S396). The data are presented as mean values ± SD (*n* ≥ 3). *p < 0.05, and ***p < 0.001.

Amounting studies have demonstrated that AD pathologies including synaptic loss and abnormal tau hyperphosphorylation were induced by STZ (Planel et al., 2007; Chen et al., 2013; Kamat et al., 2016; Omidi et al., 2020). To assess the STZ effect on the synaptic function of hippocampus cells, we verified the PDS95 protein level, a post-synapse marker using immunoblotting. The results indicated that the PSD95 level was reduced by STZ in a time-dependent manner (Figure 2A). We also showed that phosphorylation of tau epitopes, such as AT8(S202/T205) and p-Tau(S262) was significantly up-regulated by STZ treatment, whereas p-Tau(T181) and p-Tau(S396) were unchanged (Figure 2B). These results indicated that STZ treatment on HT-22 cells induced sAD-like pathology, such as synaptic loss and tauopathy, in hippocampal neuron cells.

### STZ-Induced Drp1-Mediated Mitochondrial Fragmentation

**Figure 3:**
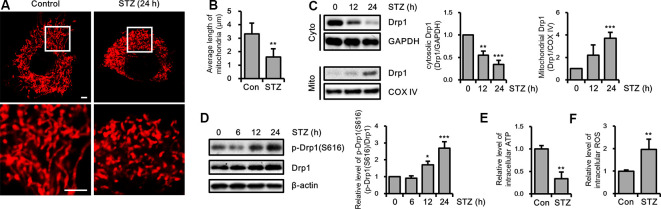
Effect of streptozotocin (STZ) on changes in Drp1-related mitochondrial morphology. **(A)** Mitochondrial morphology in DsRed2-mito-expressing control and STZ-treated HT-22 cells after 24 h were observed using confocal microscopy. The bottom panels showed the magnified images of regions indicated by white squares in the top panels; scale bar, 5 μm. **(B)** The graphs showed an average mitochondrial length. **(C)** Cytoplasm and mitochondrial fractions of HT-22 cells treated with STZ for the indicated time were analyzed by western blotting using antibodies against Drp1, COXIV, and GAPDH. GAPDH and COXIV were used as the loading control for the cytoplasm and mitochondria, respectively. **(D)** The levels of p-Drp1(S616) were determined by western blot analysis in STZ-treated HT-22 cells. Drp1 was the loading control of p-Drp1(S616). **(E)** Intracellular adenosine triphosphate (ATP) levels in STZ-treated HT-22 cells for 24 h were measured using the ATP determination kit. **(F)** Flow cytometry analysis of intracellular reactive oxygen species (ROS) levels in STZ-treated HT-22 cells for 24 h was analyzed with CM-H2DCFDA (5 μM). The data are presented as mean values ± SD (*n* ≥ 3). *p < 0.05, **p < 0.01, and ***p < 0.001.

To determine whether STZ affects mitochondrial dynamics, we evaluated changes in mitochondrial morphology by STZ in mitochondria-targeting DsRed2 (DsRed2-mito) stably expressing HT-22 cells. Our result showed that punctate mitochondrial formation was increased and the mitochondrial average length was decreased in the STZ-treated HT-22 cells (Figures 3A,B). Drp1 recruited from the cytosol to mitochondria, then triggers mitochondrial division (Westermann, 2010; Korobova et al., 2013). To assess whether STZ treatment affected Drp1 localization, we isolated cytoplasmic and mitochondrial proteins; then we analyzed the levels of Drp1 *via* immunoblotting. Our results indicated that cytoplasmic Drp1 was decrease and mitochondrial Drp1 was increased after STZ treatment for 12 h (Figure 3C). Phosphorylation of Drp1 at S616 has been shown to control activity and localization of Drp1 from the cytosol to mitochondria (Prieto et al., 2016). An increase in the phosphorylation of Drp1 at S616 has also been observed in patients with AD (Wang et al., 2009). Accordingly, we evaluated for changes in the phosphorylation level of Drp1 at S616 after STZ treatment in a time-dependent manner *via* western blotting analysis. As expected, the levels of phosphorylated Drp1 at S616 was dramatically increased after STZ treatment for 12 h without any changes in other mitochondrial fission and fusion proteins including Drp1, Fis1, Opa1, Mfn1, and Mfn2 (Figure 3D and Supplementary Figure S1). We also measured changes in intracellular ATP and ROS levels after STZ treatment since STZ is also known to affect mitochondrial function. Our results showed that STZ-treated HT-22 cells had lower intracellular ATP levels and higher intracellular ROS levels compared to control cells (Figures 3E,F). These results suggested that STZ triggered the Drp1 translocation from the cytoplasm to the mitochondria by phosphorylation of Drp1 at S616, resulting in fragmented and damaged mitochondria.

### Effects of Mitochondria Fragmentation on STZ-Induced sAD-Like Pathology

**Figure 4:**
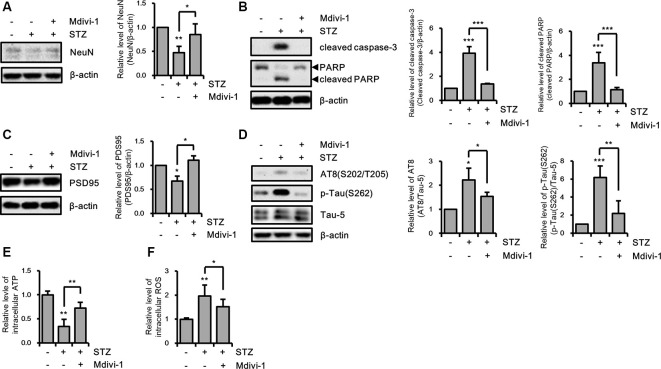
The effects of streptozotocin (STZ)-induced mitochondrial fission on Alzheimer’s disease (AD)-like pathology of HT-22 cells. **(A)** The protein levels of NeuN, **(B)** cleaved caspase-3, PARP, **(C)** PSD95, **(D)** AT-8, and p-Tau(S262) were determined by western blotting in STZ-treated HT-22 cells in the presence or absence of Mdivi-1 (12.5 μM). **(E,F)** The effect of Mdivi-1 on intracellular ATP and ROS levels was analyzed using the ATP determination kit and CM-H2DCFDA (5 μM), respectively in STZ-treated HT-22 cells at 24 h. The data are presented as mean values ± SD (*n* ≥ 3). *p < 0.05, **p < 0.01, and ***p < 0.001.

Excessive mitochondrial fission can affect various cellular processes in hippocampal neurons (Kim B. et al., 2016; Jiang et al., 2018). Thus, we investigated the effect of STZ-induced mitochondrial fragmentation on hippocampal cell loss, synaptic function, and tauopathy using the mitochondrial fission inhibitor Mdivi-1, which inhibits Drp1 GTPase activity (Cassidy-Stone et al., 2008). The results revealed that the reduced expression level of NeuN by STZ was rescued by Mdivi-1 treatment (Figure 4A). Media-1 suppressed the increased levels of cleaved caspase-3 and cleaved PARP due to the STZ treatment (Figure 4B). Also, down-regulated PSD95 was repressed by Mdivi-1 (Figure 4C). The STZ-induced increased levels of AT8 and p-Tau(S262) were attenuated by Midiv-1 (Figure 4D). Deficient intercellular ATP and increased ROS levels by STZ were also restored by inhibition of mitochondrial fission (Figures 4E,F). Consequently, these results indicated that STZ-mediated mitochondrial dysfunction induced by excessive mitochondrial fragmentation can influence hippocampal neuronal loss, synaptic function, and tau activation.

### Influence of CDK5 on STZ-Induced Mitochondrial Fission

**Figure 5:**
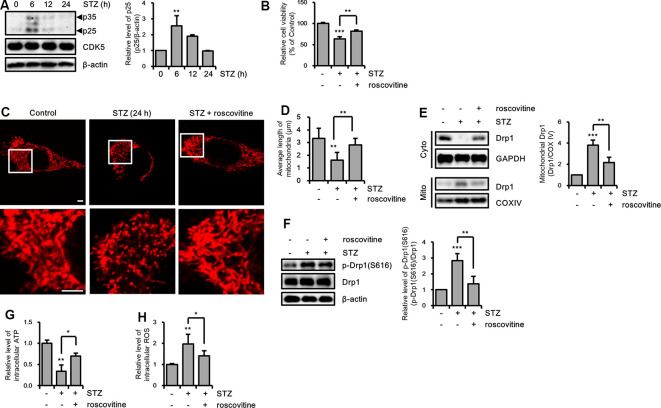
The effect of CDK5 on streptozotocin (STZ)-induced Drp1-mediated mitochondrial fission.** (A)** Protein levels of p35/25 and CDK5 in STZ-treated HT-22 cells were determined by western blotting. **(B)** Cell viability of HT-22 cells treated with STZ for 24 h was determined in the presence or absence of roscovitine (10 μM). **(C)** Changes in mitochondrial morphology were observed using confocal microscopy in DsRed2-mito expressing HT-22 cells after STZ treatment for 24 h with or without roscovitine. The bottom panels show the magnified images of regions indicated by white squares in the top panels; scale bar, 5 μm. **(D)** The graphs show the average mitochondrial length. **(E)** Cytoplasmic and mitochondrial Drp1 localization was characterized by cytoplasm and mitochondrial protein fractions of HT-22 cells treated with STZ for 24 h in the presence or absence of roscovitine. GAPDH and COX IV were used as loading controls for the cytoplasm and mitochondria, respectively. **(F)** Proteins level of p-Drp1(S616) in STZ-treated HT-22 cells with or without roscovitine at 24 h. Drp1 was the loading control of p-Drp1 at S616. **(G)** The intracellular level of ATP was measured in STZ-treated HT-22 cells with or without roscovitine. **(H)** The intracellular ROS was measured in STZ-treated HT-22 cells at 24 h in the presence or absence of roscovitine. The data are presented as mean values ± SD (*n* ≥ 3). *p < 0.05, **p < 0.01, and ***p < 0.001.

Drp1 at S616 can be phosphorylated by kinases, such as extracellular signal-regulated kinase (ERK, also known as p42/44 MAPK), glycogen synthase kinase 3 β (GSK3β), and CDK5 (Yu et al., 2011; Huang et al., 2015; Guo et al., 2018). Thus, we determined the activity levels of these kinases by measuring the resulting phosphorylation levels. Our findings revealed that p-ERK was increased by a 12-h STZ treatment. Also, p25, which is a specific activator of CDK5, was increased by STZ at 6 h, whereas the p-GSK3β(S9) level did not significantly change (Figure 5A and Supplementary Figure S2A). Because ERK and CDK5 were increased by STZ treatment, we assessed the signaling pathway affected by phosphorylation of Drp1 at S616 using an ERK inhibitor (U0126) and CDK5 inhibitor (roscovitine). Our results indicated that inhibition of CDK5 successfully repressed STZ-induced neuronal toxicity, whereas ERK inhibition did not affect the STZ-mediated decrease in cell viability (Figure 5B and Supplementary Figure S2B). Thus, we focused on the inhibitory effect of CDK5 on STZ-mediated mitochondrial fragmentation and mitochondrial function. The results showed that the number of punctate mitochondria decreased after roscovitine treatment, and the average mitochondrial length returned to control levels (Figures 5C,D). Furthermore, STZ-induced levels of p-Drp1 at S616 and localization of Drp1 from the cytosol to mitochondria were restored by CDK5 inhibition (Figures 5E,F). CDK5 inhibition also conferred protection from damaged mitochondrial processes such as decreased levels of intracellular ATP and increased levels of intracellular ROS (Figures 5G,H).

**Figure 6:**
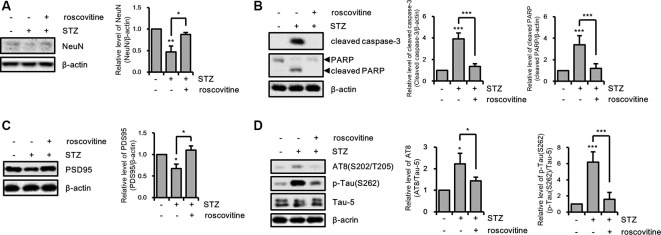
The effect of CDK5 on streptozotocin (STZ)-induced neuronal loss and tau activation.** (A)** NeuN, **(B)** cleaved caspase-3, PARP, **(C)** PSD95, **(D)** AT-8, and p-Tau(S262) levels of STZ-induced HT-22 cells treated with or without roscovitine (10 μM) after 24 h was confirmed by western blotting. The data are presented as mean values ± SD (*n* ≥ 3). **p < 0.05, **, ***p < 0.001.

We confirmed the effects of the CDK5/p25 signaling pathway on STZ-induced apoptotic neuronal loss with CDK5 inhibition. STZ-induced apoptotic neuronal loss was reversed by treatment with a CDK5 inhibitor (Figures 6A,B). Moreover, CDK5 inhibition rescued STZ-induced synaptic loss, and resulted in increased levels of AT8 and p-Tau(S262), decreased levels of intracellular ATP, and increased levels of intracellular ROS (Figures 6C,D). These results suggested that STZ-induced sAD-like pathology in HT-22 cells were involved in CDK5-mediated Drp1-dependent mitochondrial fragmentation.

## Discussion

Previously, we developed an effective and clinically relevant non-human primate sAD-like model using cisterna magna (CM)-STZ injection; the model is characterized by cerebral damage, the disintegration of the neurovascular unit, neuroinflammation, Aβ deposition, and hippocampal neuronal loss (Yeo et al., 2015). However, the molecular mechanisms underlying the STZ-induced sAD-like monkey model remained unknown.

To study the mechanistic details specific to the STZ-induced sAD processes, we constructed an STZ-mediated cellular model in HT-22 cells, which are immortalized mouse hippocampal neuronal cells (Davis and Maher, 1994). We previously reported hippocampal neuronal toxicity in HT-22 cells treated with Aβ and iron, which are both considered causative agents of AD (Park et al., 2015b; Kim B. et al., 2016).

Our STZ-induced HT-22 cellular model successfully reflected sAD-like pathologies, such as neuronal apoptosis, synaptic loss, and tau phosphorylation. Previously, some investigations were performed to identify the effects of STZ on neuronal cell lines, such as Neuro-2A cells and SH-SY5Y, respectively (Biswas et al., 2016; Guo et al., 2017). To discover the effect of STZ on neurotoxic and inducing sAD-like pathology in hippocampal neurons, we observed the effect of STZ in the hippocampal neuronal cell line, HT-22 cells. STZ treatment cellular model was suggested with drug screening for AD therapeutic target. Therefore, more information for the molecular signal pathway was demanded to find the diverse molecular therapeutic target. Therefore, we focused on the change in mitochondrial morphology and involved molecular pathways.

Mitochondria dysfunction is an early driver of AD, and the brains of patients with AD show defective energy metabolism (Reddy, 2009; Rhein et al., 2009). Patients and cellular models of AD show an abnormal balance in mitochondrial dynamics (Wang et al., 2008; Cha et al., 2012; DuBoff et al., 2013). Also, impairment of mitochondrial dynamics was exhibited in an STZ-induced diabetic mice brain (Correia et al., 2013; Chen et al., 2014; Santos et al., 2014; Zhou et al., 2018). Furthermore, inhibition of mitochondrial dysfunction restored STZ-induced memory deficit and impaired neuronal function (Li et al., 2017; Omidi et al., 2020). In the current study, we discovered that mitochondrial fission was increased in STZ-treated HT-22 cells. To verify whether impaired homeostasis of mitochondrial dynamics affected STZ-induced sAD-like pathologies, we used Mdivi-1, which is a chemical inhibitor of mitochondrial fission. Our results indicated that STZ-induced neuronal loss, apoptosis, synaptic loss, tau phosphorylation, and impaired mitochondrial function were attenuated by Mdivi-1 treatment. Further evidence suggested that inhibition of mitochondrial fission by Mdivi-1 ameliorated mitochondrial dysfunction and conferred protection from neuronal cell death (Rappold et al., 2014; Kim H. et al., 2016; Reddy et al., 2017). Therefore, the regulation of mitochondrial dynamics may be a potential therapeutic strategy for AD.

Because the past study showed that Drp1 and Fis1, mitochondrial fission proteins, were increased in the AD mice model (Wang et al., 2008), we confirmed total protein expression levels of mitochondrial dynamics factors to identify alternative key regulators related to STZ-induced mitochondrial fission. Our results indicated that Drp1 and Fis1 were unchanged by STZ treatment; whereas translocation of Drp1 to mitochondria was increased through phosphorylation of Drp1 at S616. It was similar results to the earlier study demonstrated that Drp1 location was more important than the total expression level in the AD cellular model (Joshi et al., 2018). Also, the result of increasing phosphorylation of Drp1 at S616 was consistent with past studies in the brains of AD model mice and cells (Wang et al., 2009; Dowding et al., 2014). Several studies have suggested that ERK activation leads to neuronal cell death and affected mitochondrial dynamics by regulating the phosphorylation of Drp1 (Cagnol and Chambard, 2010; Ha et al., 2012; Gan et al., 2014). GSK3β has also been shown to be related to imbalanced mitochondrial dynamics in the hippocampus of mice with type 2 diabetes and a human neuronal cell line *via* a Drp1-dependent mechanism (Huang et al., 2015). Furthermore, the deregulation of CDK5 contributes to the pathological development of AD as a regulator of mitochondrial fragmentation during neuronal apoptosis, and its suppression attenuates apoptotic excessive mitochondrial fission (Cruz et al., 2006; Giese, 2014; Guo et al., 2018). Therefore, we confirmed the changes in the activation levels of ERK, GSK3β, and CDK5. ERK and CDK5 were activated by STZ treatment, but GSK3β was not dramatically activated.

In STZ-induced cognitive impairment models, changes in the level of p-GSK3β(S9) has been controversial. Several studies have indicated that the level of p-GSK3β(S9) was increased in ICV-STZ injection models (Chen et al., 2013; Zhu et al., 2019), but decreased in intraperitoneal-STZ injections models (Clodfelder-Miller et al., 2006; Gratuze et al., 2017). Also, similar to our results, some studies have shown that the STZ-induced level of p-GSK3β(S9) was not changed (Chen et al., 2013; Guo et al., 2017). Therefore, we excluded GSK3β as a molecular target against STZ-induced sAD-like pathology in the hippocampus cell line.

We investigated the protective effect of ERK and CDK5 on STZ-induced neuronal toxicity using their chemical inhibitors, respectively. Our results showed that ERK inhibition did not affect STZ-induced hippocampal toxicity, while it was significantly rescued by CDK5 inhibition. ERK and CDK5 activation have been closely implicated with the progression of AD pathology and unbalanced mitochondrial dynamics, and inhibition of these proteins have been regarded as a potential strategy for AD therapeutics (Cruz et al., 2006; Cagnol and Chambard, 2010; Ha et al., 2012; Gan et al., 2014; Giese, 2014). Our study indicated that the ERK signaling pathway was activated by Aβ oligomer stimulation in HT-22 cells, and ERK controlled neuronal apoptosis and led to excessive mitochondrial fragmentation (Kim B. et al., 2016). Although ERK activation was also observed in an STZ-mediated AD model (Liu et al., 2019), some studies have indicated that p-ERK was not altered by STZ-injection. CDK5 closely interacts with Drp1 activation through phosphorylation of Drp1(S616), and these interactions affect the various neuronal process, such as neuronal maturation (Cho et al., 2014). Furthermore, dysregulated CDK5 phosphorylates Drp1 and induces mitochondrial fragmentation, neuronal apoptosis, and cell death, and its inhibition protects from various neuronal toxins (Jahani-Asl et al., 2015; Guo et al., 2018). Previous results including our study in the CM-STZ injected non-human primate AD brain showed various cases of STZ-mediated changes in CDK5, p35, and p25 levels (Clodfelder-Miller et al., 2006; Chen et al., 2013; Park S. J. et al., 2015; Gratuze et al., 2017). Therefore, our results indicated that CDK5 affected Drp1 activation and mitochondrial fission *via* phosphorylation of Drp1(S616) in the STZ-induced AD-like cellular model.

These diverse molecular signals in a model of STZ-mediated cognitive deficit seemed to result from methodic differences such as the injection strategy, experimental period, and analytic type of cells in the brain. Furthermore, almost all studies utilizing STZ have been confirmed with hippocampal brain tissue, which included a mixed population of cells such as oligodendrocytes, microglia, astrocytes, and neurons. Therefore, we tried to identify molecular mechanistic features and molecular therapeutic targets in an AD conditional hippocampal neuronal cell line induced by STZ treatment. Furthermore, we screened the STZ-induced molecular signal pathway in a time-dependent manner. Our findings indicated that CDK5 activation occurred before phosphorylation of Drp1 at S616, while ERK activation was generated at a similar time point.

These results were different from other studies using neuroblastoma cells, which showed AD hallmarks such as neuronal loss, synaptic loss, tau phosphorylation, mitochondrial fission, and ROS generation by STZ treatment. However, the STZ-induced molecular signaling pathway was different from our results. Therefore, to acquire more precise information, further investigations in conditions more similar to those of the brain, such as primary cultured astrocytes, microglia, and neuron cells from the hippocampus, are needed. Collectively, we suggested that the STZ-induced up-regulator CDK5 and changes in mitochondrial morphology may be an important factor and marker in sAD-like processes in hippocampus neurons.

Using an STZ-mediated cellular model reflecting sAD-like pathology in HT-22 cells, we found that STZ-induced early activation of CDK5 was related to Drp1-dependent mitochondrial fragmentation, and CDK5 was recognized as a key regulator for STZ-induced AD-like pathology. Our results revealed that CDK5 is an essential regulator of STZ-induced sAD-like pathology by playing a key role in regulating mitochondrial fission involved in hippocampal neurodegeneration. Based on these results, we will verify the molecular mechanism focusing on the CDK5-Drp1 pathway by applying to our previous AD-like model (Park S. J. et al., 2015; Yeo et al., 2015). Consequently, regulation of mitochondrial morphology and function may be a potential inhibitor of neurodegeneration triggered by CDK5/Drp1-dependent metabolic impairment and may represent a possible strategy for developing therapies to treat abnormal metabolic functions associated with the pathogenesis of sAD.

## Data Availability Statement

The raw data supporting the conclusions of this article will be made available by the authors, without undue reservation.

## Author Contributions

JP participated in the design of the experiments, performed the experiments, analyzed the data, and drafted the manuscript. JW, JS, H-GY, KK, YK, C-YJ, and MK performed and supported the experiments. Y-HK, J-WH, S-RL, D-SL, and YL edited and revised the manuscript.

## Conflict of Interest

The authors declare that the research was conducted in the absence of any commercial or financial relationships that could be construed as a potential conflict of interest.

## Supplementary Material

FIGURE S1 The expression level of mitochondrial dynamics proteins in streptozotocin (STZ)-treated HT-22 cells. The levels of Fis1 (mitochondrial fission), Opa1, Mfn1, and Mfn2 (mitochondrial fusion) were verified by western blotting in STZ-treated HT-22 cells.

FIGURE S2 The effect of ERK in streptozotocin (STZ)-treated HT-22 cells. **(A)** The levels of p-ERK and p-GSK3β were confirmed by western blotting in streptozotocin (STZ)-treated HT-22 cells for 24 h. ERK and GSK3β were the loading controls of p-ERK and p-GSK3, respectively. **(B)** The cell viability of HT-22 cells treated with STZ for 24 h was determined in the presence or absence of U0126 (10 μM). The data are presented as mean values ± SD (*n* ≥ 3). ***p* < 0.01, ****p* < 0.001, and N.S. denotes no significant difference.

## Abbreviations

ATP: adenosine triphosphate
CO_2_: Carbon Dioxide
DMEM: Dulbecco’s Modified Eagle Medium
DMSO: Dimethyl sulfoxide
FBS: fetal bovine serum
MTT: 3-(4,5-dimethylthiazol-2-yl)-2,5-diphenyltetrazolium bromide
Mdivi-1: 3-(2,4-Dichloro-5-methoxyphenyl)-2,3-dihydro-2-thioxo-4(1H)-quinazolinone
PBS: phosphate-buffered saline
ROS: reactive oxygen species
SDS-PAGE: sodium dodecyl sulfate-polyacrylamide gel electrophoresis
TBS: tris-buffered saline.
