# Supplementary figures and images for: Streptozotocin Induces Alzheimer’s Disease-Like Pathology in Hippocampal Neuronal Cells via CDK5/Drp1-Mediated Mitochondrial Fragmentation
Source: Front Cell Neurosci. 2020 Aug 4;14:235. doi: 10.3389/fncel.2020.00235 (PMC7438738; doi:10.3389/fncel.2020.00235)

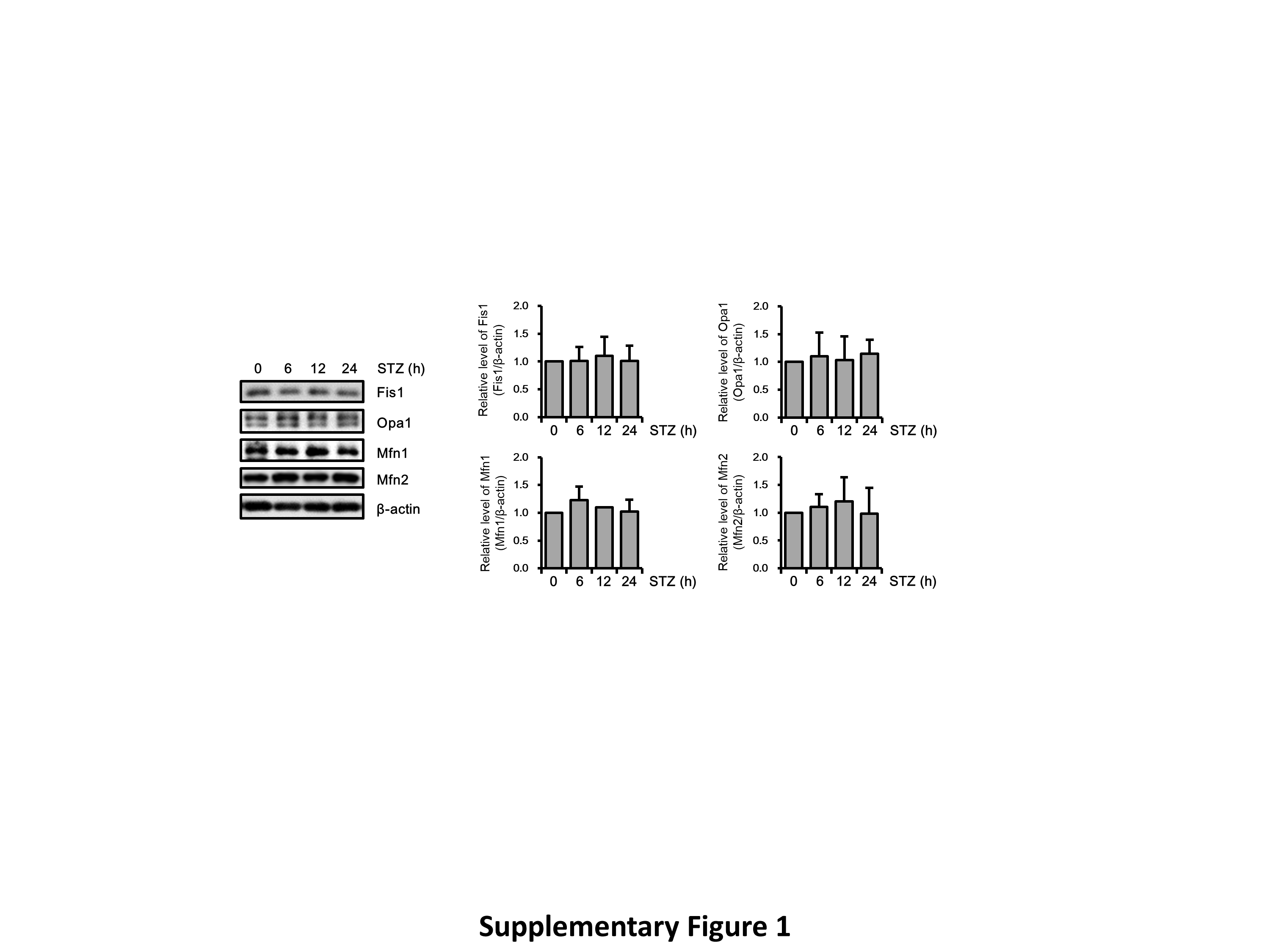

Supplement: FIGURE S1 — The expression level of mitochondrial dynamics proteins in streptozotocin (STZ)-treated HT-22 cells. The levels of Fis1 (mitochondrial fission), Opa1, Mfn1, and Mfn2 (mitochondrial fusion) were verified by western blotting in STZ-treated HT-22 cells. [file Image_1.TIF]

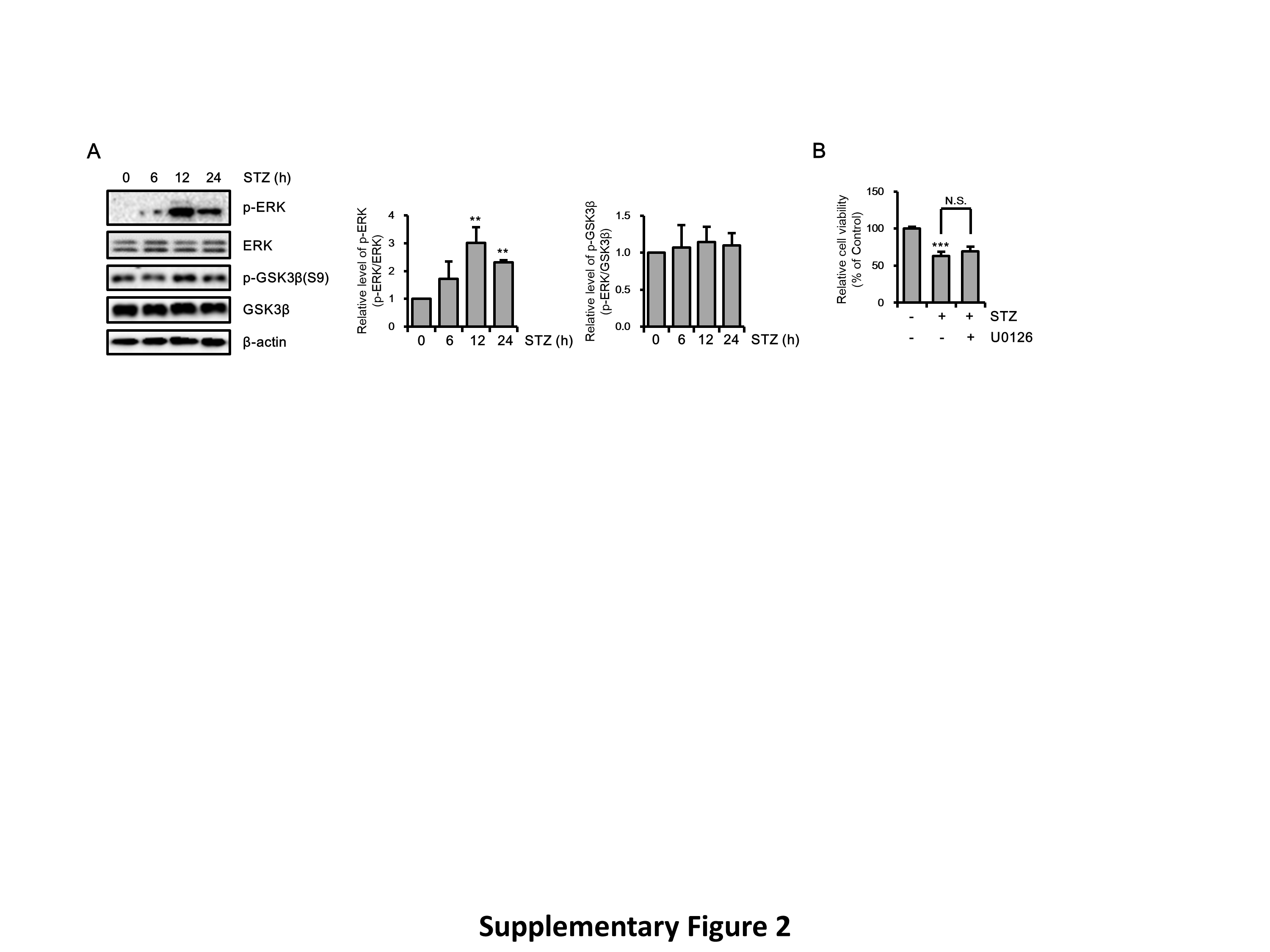

Supplement: FIGURE S2 — The effect of ERK in streptozotocin (STZ)-treated HT-22 cells. (A) The levels of p-ERK and p-GSK3β were confirmed by western blotting in streptozotocin (STZ)-treated HT-22 cells for 24 h. ERK and GSK3β were the loading controls of p-ERK and p-GSK3, respectively. (B) The cell viability of HT-22 cells treated with STZ for 24 h was determined in the presence or absence of U0126 (10 μM). The data are presented as mean values ± SD (n ≥ 3). **p < 0.01, ***p < 0.001, and N.S. denotes no significant difference. [file Image_2.TIF]
